# Generalized Multiphase Dynamic Modeling and Precision Interaction Force Control of a Walking Lower Limb Hydraulic Exoskeleton

**DOI:** 10.1155/2022/2801719

**Published:** 2022-04-07

**Authors:** Shan Chen, Muye Lu, Fangfang Dong, Haijun Liu, Xiaoqing Tian, Jiang Han

**Affiliations:** ^1^School of Mechanical Engineering, Hefei University of Technology, Hefei 230009, China; ^2^Anhui Engineering Laboratory of Intelligent CNC Technology and Equipment, Hefei 230009, China; ^3^HMB Research Institute of Automation and Intelligent Equipment, Ma'anshan 243131, China

## Abstract

Wearable lower limb hydraulic exoskeletons can be used to augment the human performance in heavy load transportation. Nonlinear and walking phase-dependent dynamics make the lower limb hydraulic exoskeleton become difficult to be modeled. This paper presents a generalized multiphase dynamic modeling method in which the dynamic model of each walking phase can all be solved based on a general higher dimensional dynamic model and different holonomic constraints. Compared to traditional lower limb exoskeleton modeling methods where the modeling of each walking phase is done independently, the proposed method is simple and applicable to arbitrary walking phases, especially for double leg support phase (closed-chain dynamics). Based on the established dynamic models, MIMO adaptive robust cascade force controllers (ARCFC) are designed both for double leg support phase and single leg support phase to effectively address high-order nonlinearities and various modeling uncertainties in hydraulic exoskeletons. An additional torque allocation method is proposed to deal with the overactuated characteristic in double leg support. Comparative simulations are conducted to verify the excellent human-machine interaction force control performance of the proposed scheme.

## 1. Introduction

Wearable lower limb exoskeletons are intelligent human-machine integrated devices used to augment the performance of the wearer in heavy load transportation [1, 2], such as soldier marching, earthquake rescue, and construction site. Thanks to the large ratio of power-to-weight, it is suitable to adopt hydraulic actuators in developing such systems which need to be a small size while providing large force. In hydraulic lower limb exoskeleton, the wearer provides motion commands, while hydraulic actuators supply enough actuation force to support heavy loads. When the exoskeleton tracks the human motion precisely, little load force can be felt by the wearer. Since the human-machine interaction force is closely related to the trajectory tracking error between the wearer and exoskeleton, the control objective can finally be transformed into minimizing the human-machine interaction force.

As for the interaction force controller design of a walking hydraulic lower limb exoskeleton system, a number of challenging issues exist. First, different from 1-DOF or single leg exoskeleton, there exist multiple walking phases in the walking lower limb exoskeleton, such single leg support phase, double leg support phase, and subphases during double leg support (like toe off and heel strike). Moreover, for double leg support phase and its subphases, the two feet are landing the ground at the same time leading to a closed-chain mechanism. Also overactuated conditions exist in closed chain dynamics. All these make the dynamic modeling and controller design of lower limb exoskeleton become more difficult. Besides, as a nondesired force output source, high-order nonlinearities and various model uncertainties exist in hydraulic actuators which make the controller design of hydraulic lower limb exoskeleton more challenging than that of motor-driven system.

As for the multiphase dynamic modeling of lower limb exoskeletons, usually the dynamic model of each walking phase is established separately. For single leg support phase, since it is a serial-chain mechanism, the dynamic model is usually obtained straightforwardly using Lagrangian equations. In [3], the lower limb exoskeleton in single leg support is described as serial chain of 7 segments. Using Lagrangian equations of the second kind, the planar model of single leg support is obtained. In [4], the Lagrange equations of the second kind is adopted to model the single leg support phase of 6-link biped robot. For double leg support phase or its subphases (such as toe off and heel strike), since it is a closed-chain mechanism with overactuation, the Lagrangian of the second kind cannot be applied directly. In [3], the lower limb exoskeleton in double leg support is partitioned into two 3-DOF serial manipulators. Lagrangian equations of the second kind are used to model the dynamics of each 3-DOF manipulator, while Newtonian mechanics is adopted to describe the relationship of the two parts. In [4], the Lagrange formulation of the first kind using Lagrange multipliers is adopted for dynamic modeling of biped robot in double leg support. However, all these methods focus on the dynamic modeling of each walking phase independently in which different general coordinates needed to be established and different Lagrangian modeling process need to be conducted for each walking phase. This obviously leads to a complicated modeling process when there exist various walking phases. Also, the joint positions may become discontinuous due to the role switching of the swing and support leg [5].

As for the human-machine interaction force controller design, various control schemes are proposed [6]. One method is to design the exoskeleton controller without measuring the human-machine interaction force. In [7], an inverse dynamics-based positive feedback controller is designed for the Berkeley lower extremity exoskeleton so that the sensitivity to the human force can be increased. In [8], first the inverse dynamics of the exoskeleton is adopted to estimate the joint torque, and then, a fictitious gain is designed to increase the sensitivity of the human body. However, these methods ignore the model uncertainties in computing the inverse dynamics leading to a poor robust performances. Another line of thought is to design a cascade force controller based on the measured human-machine interaction force. Model-free PID controller [9], admittance controller [10], and human-machine cooperation controller [9] are often used in the high-level control algorithm design to generate the desired motion command, while PID [11] or dynamic model compensation are often used in the low-level controller design to achieve the motion tracking. In these cascade force control schemes, the human motion intent is inferred from the fixed dynamic model or human data which cannot be generalized to different wearers. Also, the proposed low-level motion tracking algorithm cannot guarantee the fast response and accurate tracking of human motion in the presence of strongly coupled nonlinearities and various model uncertainties. Other control methods have considered the model uncertainties in the controller design, such as the adaptive impedance control [12], neural network control [13], sliding mode controller [14, 15], and robust output feedback-assistive control [16]. However, these methods are mainly focused on the controller design of motor-driven exoskeletons which cannot be easily extended to the control of hydraulic exoskeletons. The reason is that the order of hydraulic exoskeleton dynamics is higher (it is a three-order dynamics for hydraulic exoskeleton in this paper, while it is usually a two-order dynamics for motor-driven exoskeleton systems). Also, various model uncertainties exist in the hydraulic actuators, like the load change, hydraulic parameters variation (e.g., bulk modulus), external disturbances, and leakage.

Recently, an adaptive robust cascade force control algorithm is proposed for 1-DOF and 3-DOF hydraulic exoskeleton system [17, 18]. Robust interaction force control performance to various model uncertainties is guaranteed. In this paper, the problem is extended to a walking lower limb exoskeleton. Compared to the 3-DOF single leg exoskeleton, multiple walking phases exist when two legs move, which makes the human motion intent inference method of lower limb exoskeleton different from that of single leg exoskeleton. Moreover, the closed-chain dynamics and overactuated characteristic in double leg support and its subphases make the interaction force controller design become more challenged.

This paper presents a generalized multiphase dynamic modeling method and a robust interaction force control scheme for hydraulic lower limb exoskeleton. The principal contributions can be summarized as follows:

1. A generalized multiphase dynamic modeling method is proposed for lower limb exoskeleton which is applicable to arbitrary walking phase, especially for double leg support phase (closed-chain dynamics). Since the generalized coordinates are the same and the Lagrangian modeling process will only be done once, the multiphase dynamics modeling process of a walking lower limb exoskeleton becomes simple
2. Multiphase adaptive robust interaction force controllers are designed to effectively deal with overactuated characteristic, negative effect of high-order nonlinearities of hydraulic system, various parameter uncertainties, and modeling errors. Robust interaction force control performance is achieved

## 2. Physical Modeling

Since human dynamics is very complicated and may not be conveniently applied for exoskeleton controller design, in our modeling, we do not establish the human model, and the humans are just regarded to provide a desired motion trajectory. Then, a human-machine interface dynamic model is established to describe the relationship between interaction force and human-exoskeleton motion tracking error. The whole dynamic model of hydraulic lower limb exoskeleton includes three parts: rigid body dynamics of lower limb exoskeleton, hydraulic actuator dynamics, and human-machine interface dynamics.

### 2.1. Rigid Body Dynamics

#### 2.1.1. General Higher Dimensional Dynamic Model

**Figure 1:**
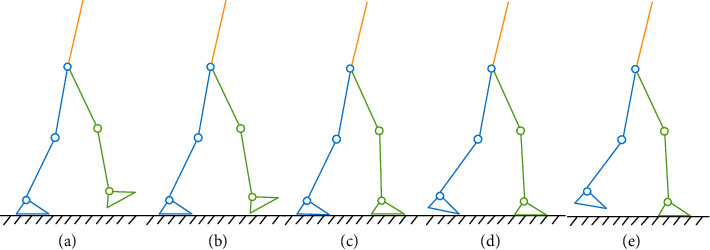
Multiple walking phases. (a) Left leg support. (b) Right heel strike. (c) Double leg support. (d) Left toe off. (e) Right leg support.

In this article, a walking lower limb hydraulic exoskeleton in the sagittal plane with six fully actuated revolute joints is considered. During the walking process, there are five typical walking phases, which are left leg support phase, right heel strike phase, double leg support phase, left toe off phase, and right leg support phase, as shown in Figure 1.

**Figure 2:**
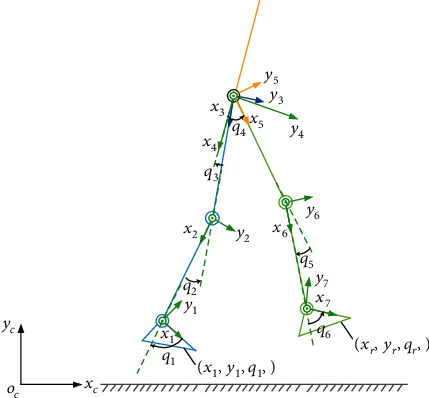
Coordinate frames for a floating exoskeleton (with positions of the exoskeleton feet changed). (*x*_*l*_, *y*_*l*_, *q*_*l*_) and (*x*_*r*_, *y*_*r*_, *q*_*r*_) represent the positions of the left foot and right foot in the Cartesian coordinate frame, receptively.

For a floating exoskeleton in which positions of the two exoskeleton feet can be changed, as shown in Figure 2, we can define nine generalized coordinates to completely describe the positions of such system. Denote

(1) $$\begin{array}{c} \begin{array}{c} q={\left[x_{l}\;y_{l}\;q_{l}\;q_{1}\;q_{2}\;q_{3}\;q_{4}\;q_{5}\;q_{6}\right]}^{T},\\ T_{act}={\left[\begin{array}{cccccc} \tau_{1} & \tau_{2} & \tau_{3} & \tau_{4} & \tau_{5} & \tau_{6} \end{array}\right]}^{T},\\ \begin{array}{c} F_{L}={\left[\begin{array}{ccc} F_{xl} & F_{yl} & T_{l} \end{array}\right]}^{T},\\ F_{R}={\left[\begin{array}{ccc} F_{xr} & F_{yr} & T_{r} \end{array}\right]}^{T}, \end{array} \end{array} \end{array}$$

where *x*_*l*_, *y*_*l*_, and *q*_*l*_ represent the left foot positions in the Cartesian coordinate frame, *q*_1_ ~ *q*_6_ represent the joint positions in the left leg and the right leg, *τ*_1_ ~ *τ*_6_ represent the joint torque from the actuators, and *F*_*L*_ and *F*_*R*_ represent ground contact force at the left and right foot.

Using Lagrangian equations, the dynamic model of the above floating exoskeleton can be described as

(2) $$\begin{array}{c} M\left(q\right)\ddot{q}+C\left(q,\dot{q}\right)\dot{q}+G\left(q\right)=B_{a}T_{\text{act}}+J_{L}^{T}F_{L}+J_{R}^{T}F_{R}, \end{array}$$

where *M*(*q*), $C\left(q,\dot{q}\right)$, and *G*(*q*) represent the system matrices and gravity force, *B*_*a*_ represents the joint-torque projection matrix, and *J*_*L*_ and *J*_*R*_ represent the Jacobian matrix in the left and right foot.

#### 2.1.2. Dynamic Model for Each Walking Phase

In different walking phases, the contact condition between the exoskeleton foot and the ground is different which leads to different holonomic constraints. Combining the same general dynamic model (Equation (2)) with different holonomic constraints, we can finally solve the detailed dynamic model for each walking phase through solving amount of linear equations. It should be noted that the generalized coordinates and the general high dimensional dynamic model are the same for the modeling of each walking phase which are quite different from the traditional multiphase dynamic modeling method where the generalized coordinates will be redefined and different Lagrangian modeling process will be conducted in each walking phase.

Define the following terms:

(3) $$\begin{array}{c} \begin{array}{c} \begin{array}{c} q={\left[\begin{array}{cc} q_{1p} & q_{c} \end{array}\right]}^{T},\\ q_{lp}={\left[\begin{array}{ccc} x_{l} & y_{l} & q_{l} \end{array}\right]}^{T}, \end{array}\\ \begin{array}{c} q_{c1}={\left[\begin{array}{ccc} q_{1} & q_{2} & q_{3} \end{array}\right]}^{T},\\ q_{c2}={\left[\begin{array}{ccc} q_{4} & q_{5} & q_{6} \end{array}\right]}^{T}, \end{array}\\ \begin{array}{c} q_{c}={\left[\begin{array}{cc} q_{c1} & q_{c2} \end{array}\right]}^{T},\\ U_{1}=\left[\begin{array}{cc} I_{3\times 3} & 0_{3\times 6} \end{array}\right], \end{array}\\ \begin{array}{c} U_{2}=\left[\begin{array}{cc} 0_{6\times 3} & I_{6\times 6} \end{array}\right],\\ U_{3}=\left[\begin{array}{c} I_{3\times 3}\\ 0_{3\times 3} \end{array}\right], \end{array}\\ \begin{array}{c} U_{4}=\left[\begin{array}{c} 0_{3\times 3}\\ I_{3\times 3} \end{array}\right],\\ T_{1}=\left[\begin{array}{cc} I_{2\times 2} & 0_{2\times 1} \end{array}\right], \end{array}\\ \begin{array}{c} T_{3}=\left[\begin{array}{c} I_{4\times 4}\\ 0_{2\times 4} \end{array}\right],\\ T_{4}=\left[\begin{array}{c} 0_{4\times 2}\\ I_{2\times 2} \end{array}\right], \end{array}\\ \begin{array}{c} J_{Rq}=-{\left(J_{R}U_{1}^{T}\right)}^{-1}J_{R}U_{2}^{T},\\ J_{Rq1}=J_{R}U_{2}^{T}U_{3}, \end{array}\\ \begin{array}{c} J_{Rq2}=J_{R}U_{2}^{T}U_{4},\\ J_{Rh1}=T_{1}J_{R}U_{2}^{T}T_{3},\\ J_{Rh2}=T_{1}J_{R}U_{2}^{T}T_{4}. \end{array} \end{array} \end{array}$$

*(1) Left Leg Support Dynamics*. For left leg support, as shown in Figure 1(a), the positions and the rotation angle of the left foot are fixed. Thus, the following holonomic constraints exist

(4) $$\begin{array}{c} \begin{array}{c} \begin{array}{c} x_{l}=C_{xl},\\ y_{l}=C_{yl},\\ q_{l}=C_{ql}, \end{array} \end{array} \end{array}$$

where *C*_*xl*_, *C*_*yl*_, and *C*_*ql*_ are constant values. Besides, since the right leg is the swing one, there is no ground contact force in the right foot, namely

(5) $$\begin{array}{c} F_{R}={\left[\begin{array}{ccc} 0 & 0 & 0 \end{array}\right]}^{T}. \end{array}$$

Differentiating (4) while noting (2) and (5), we can obtain

(6) $$\begin{array}{c} \begin{array}{c} M\ddot{q}+C\dot{q}+G=B_{a}T_{\text{act}}+J_{L}^{T}F_{L}+J_{R}^{T}F_{R},\\ \begin{array}{c} {\ddot{q}}_{lp}={\left[\begin{array}{ccc} 0 & 0 & 0 \end{array}\right]}^{T},\\ F_{R}={\left[\begin{array}{ccc} 0 & 0 & 0 \end{array}\right]}^{T}. \end{array} \end{array} \end{array}$$

Here, there are 15 unknown variables ($\ddot{q}$, *F*_*L*_, and *F*_*R*_) and 15 equations (Equation (6)). Using *T*_*act*_ as input, $\ddot{q}$, *F*_*L*_, and *F*_*R*_ can be solved. Finally, the dynamics can be obtained as

(7) $$\begin{array}{c} \begin{array}{c} {\ddot{q}}_{c}=M_{\text{Lsp}}^{-1}\left(T_{\text{act}}-C_{\text{Lsp}}{\dot{q}}_{c}-G_{\text{Lsp}}\right) \end{array}, \end{array}$$

where *M*_Lsp_, *C*_*Lsp*_, and *G*_*Lsp*_ are matrices computed from M, C, and G, as follows:

(8) $$\begin{array}{c} \begin{array}{r} \begin{array}{c} M_{\text{Lsp}}=U_{2}{MU}_{2}^{T},\\ C_{\text{Lsp}}=U_{2}{CU}_{2}^{T},\\ G_{\text{Lsp}}=U_{2}G. \end{array} \end{array} \end{array}$$

*(2) Right Leg Support Dynamics*. For right leg support, the positions of the right foot are fixed. Thus, the following holonomic constraints exist:

(9) $$\begin{array}{c} \begin{array}{c} \begin{array}{c} x_{r}=C_{xr},\\ y_{r}=C_{yr},\\ q_{r}=C_{qr}, \end{array} \end{array} \end{array}$$

where *C*_*xr*_, *C*_*yr*_, and *C*_*qr*_ are constant values. Besides, since the left leg is the swing one, there is no ground contact force in the left foot, namely

(10) $$\begin{array}{c} F_{L}={\left[\begin{array}{ccc} 0 & 0 & 0 \end{array}\right]}^{T}. \end{array}$$

Since ${\left[\begin{array}{ccc} {\dot{x}}_{r} & {\dot{y}}_{r} & {\dot{q}}_{r} \end{array}\right]}^{T}=J_{R}\dot{q}$, it can be transformed into

(11) $$\begin{array}{c} {\left[\begin{array}{ccc} {\dot{x}}_{r} & {\dot{y}}_{r} & {\dot{q}}_{r} \end{array}\right]}^{T}=\left[\begin{array}{cc} J_{R}U_{1}^{T} & J_{R}U_{2}^{T} \end{array}\right]\left[\begin{array}{c} {\dot{q}}_{lp}\\ {\dot{q}}_{c} \end{array}\right]. \end{array}$$

Differentiating (9) while noting (11) and (2), we can obtain

(12) $$\begin{array}{c} \begin{array}{c} M\ddot{q}+C\dot{q}+G=B_{a}T_{act}+J_{L}^{T}F_{L}+J_{R}^{T}F_{R},\\ \begin{array}{c} {\ddot{q}}_{lp}={\dot{J}}_{Rq}{\dot{q}}_{c}+J_{Rq}{\ddot{q}}_{c},\\ F_{L}={\left[\begin{array}{ccc} 0 & 0 & 0 \end{array}\right]}^{T}. \end{array} \end{array} \end{array}$$

Here, there are 15 unknown variables ($\ddot{q}$, *F*_*L*_, and *F*_*R*_) and 15 equations (Equation (12)). Treating *T*_*act*_ as input, $\ddot{q}$, *F*_*L*_, and *F*_*R*_ can be computed. Finally, the dynamics can be obtained as

(13) $$\begin{array}{c} \begin{array}{c} {\ddot{q}}_{c}=M_{\text{Rsp}}^{-1}\left(T_{\text{act}}-C_{\text{Rsp}}{\dot{q}}_{c}-G_{\text{Rsp}}\right) \end{array}, \end{array}$$

where *M*_Rsp_, *C*_Rsp_, *G*_Rsp_, *M*_Rsp2_, *C*_Rsp2_, and *G*_Rsp2_ are matrices computed from M, C, and G, as follows:

(14) $$\begin{array}{c} \begin{array}{c} M_{Rsp}=U_{2}{MU}_{2}^{T}+J_{Rq}^{T}U_{1}{MU}_{1}^{T}J_{Rq}+U_{2}{MU}_{1}^{T}J_{Rq}+J_{Rq}^{T}U_{1}{MU}_{2}^{T},\\ C_{Rsp}=U_{2}{CU}_{2}^{T}+J_{Rq}^{T}U_{1}{CU}_{1}^{T}J_{Rq}+U_{2}{MU}_{1}^{T}{\dot{J}}_{Rq}+U_{2}{CU}_{1}^{T}J_{Rq}+J_{Rq}^{T}U_{1}{MU}_{1}^{T}{\dot{J}}_{Rq}+J_{Rq}^{T}U_{1}{CU}_{2}^{T},\\ G_{Rsp}=U_{2}G+J_{Rq}^{T}U_{1}G. \end{array} \end{array}$$

*(3) Double Leg Support Dynamics*. For double leg support, both the positions of the right foot and the left foot are fixed. Thus, the following holonomic constraints exist:

(15) $$\begin{array}{c} \begin{array}{c} \begin{array}{c} x_{l}=C_{xl},\\ y_{l}=C_{yl},\\ q_{l}=C_{ql}, \end{array}\\ \begin{array}{c} x_{r}=C_{xr},\\ y_{r}=C_{yr},\\ q_{r}=C_{qr}. \end{array} \end{array} \end{array}$$

Differentiating (15) while noting (11) and (2), we can obtain

(16) $$\begin{array}{c} \begin{array}{c} M\ddot{q}+C\dot{q}+G=B_{a}T_{\text{act}}+J_{L}^{T}F_{L}+J_{R}^{T}F_{R},\\ \begin{array}{c} {\ddot{q}}_{lp}={\left[\begin{array}{ccc} 0 & 0 & 0 \end{array}\right]}^{T},\\ {\ddot{q}}_{lp}={\dot{J}}_{Rq}{\dot{q}}_{c}+J_{Rq}{\ddot{q}}_{c}. \end{array} \end{array} \end{array}$$

Here, we have 15 unknown variables ($\ddot{q}$, *F*_*L*_, and *F*_*R*_) and 15 equations (Equation (16)). Treating *T*_act_ as input, $\ddot{q}$, *F*_*L*_, and *F*_*R*_ can be computed.

Finally, the dynamics can be obtained as

(17) $$\begin{array}{c} \begin{array}{c} {\ddot{q}}_{c1}=M_{\text{Dsp}}^{-1}\left(B_{\text{Dsp}}T_{\text{act}}-C_{\text{Dsp}}{\dot{q}}_{c1}-G_{\text{Dsp}}\right) \end{array}, \end{array}$$

where all the new matrices in (17) can be computed from M, C, and G.

Define the following terms:

(18) $$\begin{array}{c} \begin{array}{c} A_{1}=U_{3}-U_{4}J_{Rq2}^{-1}J_{Rq1},\\ A_{2}=-U_{4}^{T}+U_{4}^{T}U_{2}J_{R}^{T}{\left(U_{3}^{T}U_{2}J_{R}^{T}\right)}^{-1}U_{3}^{T},\\ A_{3}=U_{4}\left(-J_{Rq2}^{-1}{\dot{J}}_{Rq1}+J_{Rq2}^{-1}{\dot{J}}_{Rq2}J_{Rq2}^{-1}J_{Rq1}\right). \end{array} \end{array}$$

*M*_*Dsp*_, *C*_*Dsp*_, *G*_*Dsp*_ can be computed as follows:

(19) $$\begin{array}{c} \begin{array}{c} M_{Dsp}=-A_{1}^{T}U_{4}A_{2}U_{2}{MU}_{2}^{T}A_{1},\\ C_{Dsp}=-A_{1}^{T}U_{4}A_{2}\left(U_{2}{MU}_{2}^{T}A_{3}+U_{2}{CU}_{2}^{T}A_{1}\right),\\ G_{Dsp}=-A_{1}^{T}U_{4}A_{2}U_{2}G,\\ B_{Dsp}=-A_{1}^{T}U_{4}A_{2}. \end{array} \end{array}$$

*(4) Right Heel Strike Dynamics*. For right heel strike walking phase, as shown in Figure 1(b), the positions and the rotation angle of the left foot are fixed. Also, the positions of the right foot heel are fixed. Thus, the following holonomic constraints exist:

(20) $$\begin{array}{c} \begin{array}{c} \begin{array}{c} x_{l}=C_{xl},\\ y_{l}=C_{yl},\\ q_{l}=C_{ql}, \end{array}\\ \begin{array}{c} x_{r}=C_{xr},\\ y_{r}=C_{yr}, \end{array} \end{array} \end{array}$$

where *C*_*xr*_ and *C*_*yr*_ are constant values. Besides, since the right leg contact the ground on a point, there is no ground contact torque in the right foot, namely

(21) $$\begin{array}{c} T_{r}=0. \end{array}$$

Differentiating (20) while noting (2) and (11), we can obtain

(22) $$\begin{array}{c} \begin{array}{c} M\ddot{q}+C\dot{q}+G=B_{a}T_{act}+J_{L}^{T}F_{L}+J_{R}^{T}F_{R},\\ \begin{array}{c} {\ddot{q}}_{lp}={\left[\begin{array}{ccc} 0 & 0 & 0 \end{array}\right]}^{T},\\ {\ddot{x}}_{r}=0,\\ {\ddot{y}}_{r}=0. \end{array} \end{array} \end{array}$$

Here, there are 15 unknown variables ($\ddot{q}$, *F*_*L*_, and *F*_*R*_) and 15 equations (Equation (22)). Treating *T*_*act*_ as input, $\ddot{q}$, *F*_*L*_, and *F*_*R*_ can be solved.

Denote

(23) $$\begin{array}{c} \begin{array}{r} \begin{array}{c} q_{h1}={\left[\begin{array}{cccc} q_{1} & q_{2} & q_{3} & q_{4} \end{array}\right]}^{T},\\ q_{h2}={\left[\begin{array}{cc} q_{5} & q_{6} \end{array}\right]}^{T}. \end{array} \end{array} \end{array}$$

Finally, the dynamics can be obtained as

(24) $$\begin{array}{c} \begin{array}{c} {\ddot{q}}_{h1}=M_{Hs}^{-1}\left(B_{Hs}T_{\text{act}}-C_{Hs}{\dot{q}}_{h1}-G_{Hs}\right) \end{array}, \end{array}$$

where all the new matrices in (24) can be computed from M, C, and G.

Define the following terms:

(25) $$\begin{array}{c} \begin{array}{c} H_{1}=T_{3}-T_{4}J_{Rh2}^{-1}J_{Rh1},\\ H_{2}=-T_{4}^{T}+T_{4}^{T}U_{2}J_{R}^{T}{\left(T_{3}^{T}U_{2}J_{R}^{T}\right)}^{-1}T_{3}^{T},\\ H_{3}=T_{4}\left(-J_{Rh2}^{-1}{\dot{J}}_{Rh1}+J_{Rh2}^{-1}{\dot{J}}_{Rh2}J_{Rh2}^{-1}J_{Rh1}\right). \end{array} \end{array}$$

*M*_*Hs*_, *C*_*Hs*_, and *G*_*Hs*_ can be computed as follows:

(26) $$\begin{array}{c} \begin{array}{c} M_{Hs}=-H_{1}^{T}U_{4}H_{2}U_{2}{MU}_{2}^{T}H_{1},\\ C_{Hs}=-H_{1}^{T}U_{4}H_{2}\left(U_{2}{MU}_{2}^{T}H_{3}+U_{2}{CU}_{2}^{T}H_{1}\right),\\ G_{Hs}=-H_{1}^{T}U_{4}H_{2}U_{2}G,\\ B_{Hs}=-H_{1}^{T}U_{4}H_{2}. \end{array} \end{array}$$

**Figure 3:**
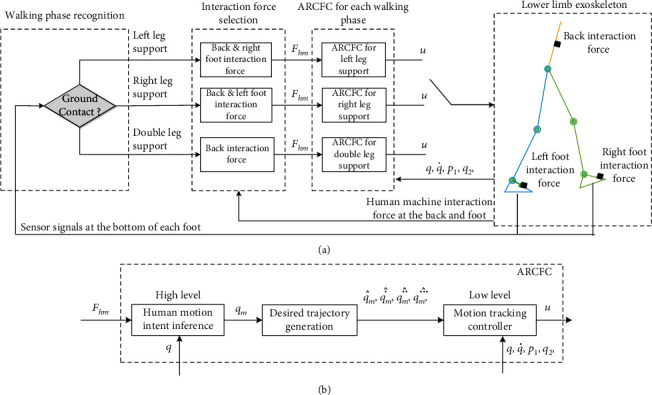
Control structure of the lower limb walking exoskeleton: (a) overall control structure and (b) structure of the adaptive robust cascade force controller (ARCFC).

*(5) Left Toe Off Dynamics*. For left toe off walking phase, shown in Figure 3(d), the positions and the rotation angle of the right foot are fixed. Also, the positions of the left foot toe are fixed. The holonomic constraints are similar to the right heel strike walking phase. Following the same computation process as that of right heel strike walking phase, the dynamic model of toe off walking phase can be obtained. The detailed computation process is omitted here for simplicity.

### 2.2. Hydraulic Actuator Dynamics

The pressure and flow rate dynamics of hydraulic cylinders can be described as [18]

(27) $$\begin{array}{c} \begin{array}{c} \frac{V_{1i}}{\beta_{e}}{\dot{P}}_{1i}=-A_{1i}\frac{\partial x_{Li}}{\partial q_{i}}{\dot{q}}_{i}+Q_{1i}+{\tilde{D}}_{31i},\\ \frac{V_{2i}}{\beta_{e}}{\dot{P}}_{2i}=A_{2i}\frac{\partial x_{Li}}{\partial q_{i}}{\dot{q}}_{i}-Q_{2i}+{\tilde{D}}_{32i},\\ \begin{array}{c} Q_{1i}=k_{q1i}x_{vi}\sqrt{\left|\Delta P_{1i}\right|},\\ Q_{2i}=k_{q2i}x_{vi}\sqrt{\left|\Delta P_{2i}\right|}, \end{array}\\ \Delta P_{1i}=\left\{\begin{array}{cc} P_{s}-P_{1i} & \text{if }x_{vi}\geq 0,\\ P_{1i}-P_{r} & \text{if }x_{vi}<0, \end{array}\right.\\ \Delta P_{2i}=\left\{\begin{array}{cc} P_{2i}-P_{r} & \text{if }x_{vi}\geq 0,\\ P_{s}-P_{2i} & \text{if }x_{vi}<0, \end{array}\right.\\ x_{vi}=u_{i},\\ i=1,\;2,\;3,\;4,\;5,\;6, \end{array} \end{array}$$

where the definition of all the terms can be seen in the notation list at the Appendix.

### 2.3. Human-Machine Interface Dynamics

The model of human-machine interaction force may be complex; for example, the model may be uncertain and/or varies. Thus, it is difficult to obtain a precise model that can describe the properties of actual human/robot attachment precisely. Also, a much precise human/robot interface dynamic model may be much complex to be used for designing the controller. Thus, in the paper, only the main property of the interface is considered. Since a belt is often adopted in connecting the robotic leg with the human leg. Thus, a spring model with unknown stiffness can describe the main compliant property of the interface. As for other unmodeled uncertainties, we put them in the lumped disturbance. In the later part, an adaptive robust control algorithm is proposed to address the modeling errors in human-machine interface dynamic model. Thus, the human-machine interface dynamic model is described as a spring with lumped disturbance and unknown stiffness:

(28) $$\begin{array}{c} \begin{array}{r} F_{hm}=K\left(x_{h}-x_{e}\right)+{\tilde{D}}_{1} \end{array}, \end{array}$$

where the definition of all the terms can be seen in the notation list at the Appendix. Equation (28) is algebraic and devoid of dynamics. Using the integral of interaction force ∫_0_^*t*^*F*_*hm*_*dτ* in the controller design, then the following dynamic model can be obtained:

(29) $$\begin{array}{c} \begin{array}{c} \frac{d}{dt}\int_{0}^{t}F_{hm}dt=K\left(x_{h}-x_{e}\right)+{\tilde{D}}_{1}. \end{array} \end{array}$$

## 3. Human-Machine Interaction Force Control Schemes

### 3.1. Control Objective

Assuming that the wearer is able to achieve balance and locomotion, the control objective of a walking lower limb exoskeleton is to design a control law according to the multi-phase dynamic models that minimizes the human-machine interaction force so that accurate human motion tracking is achieved.

### 3.2. Overall Control Structure

Figure 3(a) shows the overall control structure. Foot switches are mounted at the exoskeleton foot to recognize whether the exoskeleton foot contact the ground or not, which can further help us recognize which walking phase the exoskeleton lies in. Six-axis force sensors are fixed at the back and the foot of the lower limb exoskeleton to measure the interaction force at these places. For different walking phase, different interaction force components are selected for force controller design. Many control techniques have been developed for hydraulic systems [19–21]. The ARC is verified to be effective in addressing various model uncertainties through lots of practical applications [22–26], especially for multijoint hydraulic manipulator [27, 28]. Thus, it is adopted in the following controller design. Therefore, it will be adopted in our interaction force control algorithm design. For each walking phase, an adaptive robust cascade force controller (ARCFC) is designed so that a good robust force control performance can be achieved, as shown in Figure 3(b).

### 3.3. ARCFC Design for Double Leg Support

For the walking phase of double leg support, there are three independent degree of freedoms, which can be seen from the dynamic model (17). Thus, in double leg support, we can only control three independent human-machine interaction force components. Since the exoskeleton feet are always in flat contact with the ground and will not hinder the movement of human feet, there is no need to reduce the interaction force at the foot contact points. Finally, three human-machine interaction force components at the back are minimized. Since there are six control inputs, the system is overactuated. In the later force controller design, three independent virtual control torques are figured out first, and then, a torque allocation method is proposed to specify the desired load force for six hydraulic cylinders. Finally, six control inputs of the hydraulic valves can be figured out.

The overall system dynamics for double leg support can be described by

(30) $$\begin{array}{c} \begin{array}{c} F_{\text{hmub}}=K\left(x_{\text{hub}}-x_{\text{eub}}\right)+{\tilde{D}}_{1},\\ q_{c1}={invkine}_{\text{ub}}\left(x_{\text{eub}}\right),\\ q_{c2}={invkine}_{r}\left(x_{\text{er}},q_{c1}\right),\\ {\dot{q}}_{c2}=-J_{\text{Rq}2}^{-1}J_{\text{Rq}1}{\dot{q}}_{c1},\\ B_{\text{Dsp}}T_{\text{act}}+J_{\text{ub}}^{T}F_{\text{hmub}}=M_{\text{Dsp}}{\ddot{q}}_{c1}+C_{\text{Dsp}}{\dot{q}}_{c1}+G_{\text{Dsp}}+B{\dot{q}}_{c1}+{\tilde{D}}_{2},\\ q_{c}={\left[\begin{array}{cc} q_{c1} & q_{c2} \end{array}\right]}^{T},\\ \tau_{i}=\left(P_{1i}A_{1i}-P_{2i}A_{2i}\right)\frac{\partial x_{\text{Li}}}{\partial q_{i}},\\ \frac{V_{1i}}{\beta_{e}}{\dot{P}}_{1i}=-A_{1i}\frac{\partial x_{\text{Li}}}{\partial q_{i}}{\dot{q}}_{i}+Q_{1i}+{\tilde{D}}_{31i},\\ \frac{V_{2i}}{\beta_{e}}{\dot{P}}_{2i}=A_{2i}\frac{\partial x_{\text{Li}}}{\partial q_{i}}{\dot{q}}_{i}-Q_{2i}+{\tilde{D}}_{32i},\\ \begin{array}{c} Q_{1i}=k_{q1i}x_{\text{vi}}\sqrt{\left|\Delta P_{1i}\right|},\\ Q_{2i}=k_{q2i}x_{\text{vi}}\sqrt{\left|\Delta P_{2i}\right|}, \end{array}\\ \Delta P_{1i}=\left\{\begin{array}{cc} P_{s}-P_{1i} & \text{if }x_{\text{vi}}\geq 0,\\ P_{1i}-P_{r} & \text{if }x_{\text{vi}}<0, \end{array}\right.\\ \Delta P_{2i}=\left\{\begin{array}{cc} P_{2i}-P_{r} & \text{if }x_{\text{vi}}\geq 0,\\ P_{s}-P_{2i} & \text{if }x_{\text{vi}}<0, \end{array}\right.\\ \begin{array}{c} x_{\text{vi}}=u_{i},\\ i=1\cdots 6, \end{array} \end{array} \end{array}$$

where *K* = diag{*K*_ubx_, *K*_uby_, *K*_ubz_} is the stiffness vector of the human-machine interface and *B* = diag{*B*_1_, *B*_2_, *B*_3_} is the damping ratio. The definitions of all the other terms can be seen in the notation list at the Appendix.

The fifth equation of (30) has three properties:

**Property 1 .**

*M*_Dsp_ is a symmetric and positive definite matrix.

**Property 2 .**

${\dot{M}}_{\text{Dsp}}-2C_{\text{Dsp}}$ is a skew-symmetric matrix.

**Property 3 .**

*M*_Dsp_, *C*_Dsp_, and *G*_Dsp_ can be linearly parameterized in term of the exoskeleton system parameters *β*, i.e.,

(31) $$\begin{array}{c} \begin{array}{r} M_{\text{Dsp}}\left(q_{c}\right){\ddot{q}}_{r}+C_{\text{Dsp}}\left(q_{c},{\dot{q}}_{c}\right){\dot{q}}_{r}+G_{\text{Dsp}}\left(q_{c}\right)=f_{0}\left(q_{c},{\dot{q}}_{c},{\dot{q}}_{r},{\ddot{q}}_{r}\right)+Y\left(q_{c},{\dot{q}}_{c},{\dot{q}}_{r},{\ddot{q}}_{r}\right)\beta \end{array}, \end{array}$$

where ${\dot{q}}_{r}\text{ and }{\ddot{q}}_{r}$ are any vector and *β* is the parameter vector of the exoskeleton.

Define the following unknown parameters and lumped disturbances:

(32) $$\begin{array}{c} \begin{array}{c} \begin{array}{c} {\tilde{\Delta }}_{1}=x_{h}+K^{-1}{\tilde{D}}_{1},\\ {\tilde{\Delta }}_{3}=-{\tilde{D}}_{2}, \end{array}\\ {\tilde{\Delta }}_{4}=\left[\frac{{\tilde{D}}_{311}\beta_{e}A_{11}}{V_{11}}-\frac{{\tilde{D}}_{321}\beta_{e}A_{21}}{V_{21}},\frac{{\tilde{D}}_{312}\beta_{e}A_{12}}{V_{12}}-\frac{{\tilde{D}}_{322}\beta_{e}A_{22}}{V_{22}},\frac{{\tilde{D}}_{313}\beta_{e}A_{13}}{V_{13}}-\frac{{\tilde{D}}_{323}\beta_{e}A_{23}}{V_{23}},\frac{{\tilde{D}}_{314}\beta_{e}A_{14}}{V_{14}}-\frac{{\tilde{D}}_{324}\beta_{e}A_{24}}{V_{24}},\frac{{\tilde{D}}_{315}\beta_{e}A_{15}}{V_{15}}-\frac{{\tilde{D}}_{325}\beta_{e}A_{25}}{V_{25}},\frac{{\tilde{D}}_{316}\beta_{e}A_{16}}{V_{16}}-\frac{{\tilde{D}}_{326}\beta_{e}A_{26}}{V_{26}}\right],\\ {\tilde{\Delta }}_{i}=\Delta_{\text{in}}+\Delta_{i},\;i=1,3,4,\\ K_{\theta }={\left[\begin{array}{ccc} 1/K_{\text{ubx}} & 1/K_{\text{uby}} & 1/K_{\text{ubz}} \end{array}\right]}^{T},\\ \Delta_{1n}={\left[\begin{array}{ccc} \Delta_{1\text{ubnx}} & \Delta_{1\text{ubny}} & \Delta_{1\text{ubnz}} \end{array}\right]}^{T},\\ B_{\theta }={\left[\begin{array}{ccc} B_{1} & B_{2} & B_{3} \end{array}\right]}^{T},\\ \Delta_{3n}={\left[\begin{array}{ccc} \Delta_{3n1} & \Delta_{3n2} & \Delta_{3n3} \end{array}\right]}^{T},\\ \Delta_{4n}={\left[\begin{array}{cccccc} \Delta_{4n1} & \Delta_{4n2} & \Delta_{4n3} & \Delta_{4n4} & \Delta_{4n5} & \Delta_{4n6} \end{array}\right]}^{T},\\ \theta_{F}={\left[\begin{array}{cc} K_{\theta }^{T} & \Delta_{1n}^{T} \end{array}\right]}^{T},\\ \theta_{q}={\left[\begin{array}{ccccc} \beta^{T} & B_{\theta }^{T} & {\Delta_{3n}}^{T} & \beta_{e} & {\Delta_{4n}}^{T} \end{array}\right]}^{T},\\ \theta ={\left[\begin{array}{cc} \theta_{F}^{T} & \theta_{q}^{T} \end{array}\right]}^{T}, \end{array} \end{array}$$

where Δ_in_ and Δ_*i*_ are the constant and the time-varying part of ${\tilde{\Delta }}_{i}$. We assume that the bound of the uncertain parameters and disturbance is known.

Define the following states:

(33) $$\begin{array}{c} \begin{array}{c} x_{1}=\int_{0}^{t}F_{\text{hmub}}dt,\\ x_{2}={\left[\begin{array}{cc} x_{21} & x_{22} \end{array}\right]}^{T},\;x_{21}=q_{c1},x_{22}=q_{c2},\\ x_{3}={\left[\begin{array}{cc} x_{31} & x_{32} \end{array}\right]}^{T},\;x_{31}={\dot{q}}_{c1},x_{32}={\dot{q}}_{c2},\\ x_{4}=P_{1}={\left[\begin{array}{cccccc} P_{11} & P_{12} & P_{13} & P_{14} & P_{15} & P_{16} \end{array}\right]}^{T},\\ x_{5}=P_{2}={\left[\begin{array}{cccccc} P_{21} & P_{22} & P_{23} & P_{24} & P_{25} & P_{26} \end{array}\right]}^{T},\\ x={\left[\begin{array}{ccccc} x_{1}^{T} & {x_{21}}^{T} & {x_{31}}^{T} & {x_{4}}^{T} & {x_{5}}^{T} \end{array}\right]}^{T}. \end{array} \end{array}$$

The state space expression of dynamics (30) can be described as

(34) $$\begin{array}{c} \begin{array}{c} {\dot{x}}_{1}=-{Kx}_{\text{eub}}+K\Delta_{1n}+K\Delta_{1},\\ x_{21}={\text{invkine}}_{\text{ub}}\left(x_{\text{eub}}\right),\\ x_{22}={\text{invkine}}_{r}\left(x_{\text{er}},x_{21}\right),\\ {\dot{x}}_{21}=x_{31},\\ x_{32}=-J_{\text{Rq}2}^{-1}J_{\text{Rq}1}x_{31},\\ {\dot{x}}_{31}=M_{\text{Dsp}}^{-1}\left[B_{\text{Dsp}}{hP}_{L}+J_{\text{ub}}^{T}F_{\text{hmub}}-C_{\text{Dsp}}x_{31}-G_{\text{Dsp}}-{Bx}_{31}+\Delta_{3n}+\Delta_{3}\right],\\ {\dot{P}}_{L}=Q_{L}\beta_{e}-q_{v}x_{3}\beta_{e}+\Delta_{4n}+\Delta_{4},\\ Q_{L}=K_{q}u, \end{array} \end{array}$$

where

(35) $$\begin{array}{c} h=\mathrm{diag}\left\{\frac{\partial x_{L1}}{\partial q_{1}},\frac{\partial x_{L2}}{\partial q_{2}},\frac{\partial x_{L3}}{\partial q_{3}},\frac{\partial x_{L4}}{\partial q_{4}},\frac{\partial x_{L5}}{\partial q_{5}},\frac{\partial x_{L6}}{\partial q_{6}}\right\},\\ A_{1}=\mathrm{diag}\left\{A_{11},A_{12},A_{13},A_{14},A_{15},A_{16}\right\},\\ A_{2}=\mathrm{diag}\left\{A_{21},\;A_{22},\;A_{23},\;A_{24},\;A_{25},A_{26}\right\},\\ P_{L}=A_{1}x_{4}-A_{2}x_{5},\\ Q_{L}={\left[Q_{11}\frac{A_{11}}{V_{11}}+Q_{21}\frac{A_{21}}{V_{21}},Q_{12}\frac{A_{12}}{V_{12}}+Q_{22}\frac{A_{22}}{V_{22}},Q_{13}\frac{A_{13}}{V_{13}}+Q_{23}\frac{A_{23}}{V_{23}},Q_{14}\frac{A_{14}}{V_{14}}+Q_{24}\frac{A_{24}}{V_{24}},Q_{15}\frac{A_{15}}{V_{15}}+Q_{25}\frac{A_{25}}{V_{25}},Q_{16}\frac{A_{16}}{V_{16}}+Q_{26}\frac{A_{26}}{V_{26}}\right]}^{T},\\ q_{v}=\mathrm{diag}\left\{\left(\frac{A_{11}^{2}}{V_{11}}+\frac{A_{21}^{2}}{V_{21}}\right)\frac{\partial x_{L1}}{\partial q_{1}},\left(\frac{A_{12}^{2}}{V_{12}}+\frac{A_{22}^{2}}{V_{22}}\right)\frac{\partial x_{L2}}{\partial q_{2}},\left(\frac{A_{13}^{2}}{V_{13}}+\frac{A_{23}^{2}}{V_{23}}\right)\frac{\partial x_{L3}}{\partial q_{3}},\left(\frac{A_{14}^{2}}{V_{14}}+\frac{A_{24}^{2}}{V_{24}}\right)\frac{\partial x_{L4}}{\partial q_{4}},\left(\frac{A_{15}^{2}}{V_{15}}+\frac{A_{25}^{2}}{V_{25}}\right)\frac{\partial x_{L5}}{\partial q_{5}},\left(\frac{A_{16}^{2}}{V_{16}}+\frac{A_{26}^{2}}{V_{26}}\right)\frac{\partial x_{L6}}{\partial q_{6}}\right\},\\ K_{q}=\mathrm{diag}\left\{k_{q11}\frac{A_{11}}{V_{11}}\sqrt{\left|\Delta P_{11}\right|}+k_{q21}\frac{A_{21}}{V_{21}}\sqrt{\left|\Delta P_{21}\right|},k_{q12}\frac{A_{12}}{V_{12}}\sqrt{\left|\Delta P_{12}\right|}+k_{q22}\frac{A_{22}}{V_{22}}\sqrt{\left|\Delta P_{22}\right|},k_{q13}\frac{A_{13}}{V_{13}}\sqrt{\left|\Delta P_{13}\right|}+k_{q23}\frac{A_{23}}{V_{23}}\sqrt{\left|\Delta P_{23}\right|},k_{q14}\frac{A_{14}}{V_{14}}\sqrt{\left|\Delta P_{14}\right|}+k_{q24}\frac{A_{24}}{V_{24}}\sqrt{\left|\Delta P_{24}\right|},k_{q15}\frac{A_{15}}{V_{15}}\sqrt{\left|\Delta P_{15}\right|}+k_{q25}\frac{A_{25}}{V_{25}}\sqrt{\left|\Delta P_{25}\right|},k_{q16}\frac{A_{16}}{V_{16}}\sqrt{\left|\Delta P_{16}\right|}+k_{q26}\frac{A_{26}}{V_{26}}\sqrt{\left|\Delta P_{26}\right|}\right\}. \end{array}$$

The control goal is to synthesize a control input $u={\left[\begin{array}{cccccc} u_{1} & u_{2} & u_{3} & u_{4} & u_{5} & u_{6} \end{array}\right]}^{T}$ based on (34) that minimizes the human-machine interaction force *F*_*hmub*_.

#### 3.3.1. High-Level Human Motion Intent Inference

Treating *x*_eub_ as virtual control input, then a control law making the integral of force tracking error *z*_1_ = *x*_1_ − *x*_1*d*_ converge to zero or to be bounded is designed as

(36) $$\begin{array}{c} \begin{array}{c} x_{m}=x_{\text{ma}}+x_{\text{ms}},\\ x_{\text{ma}}=-{\hat{K}}_{f}{\dot{x}}_{1d}+{\hat{\Delta }}_{1n}=-f_{\theta_{F}}-Y_{\theta_{F}}{\hat{\theta }}_{F},\\ x_{\text{ms}}=K_{1}z_{1}+x_{\text{msn}},\\ {\dot{\hat{\theta }}}_{F}=\text{Proj}\left(-\Gamma_{1}Y_{\theta_{F}}^{T}z_{1}\right),\\ {\text{Proj}}_{i}\left({•}_{i}\right)=\left\{\begin{array}{cc} 0 & \text{if }{\hat{\theta }}_{Fi}=\theta_{F\max i}\;\text{and}\;{•}_{i}>0,\\ 0 & \text{if }{\hat{\theta }}_{Fi}=\theta_{F\min i}\;\text{and}\;{•}_{i}<0,\\ {•}_{i} & \text{otherwise}, \end{array}\right. \end{array} \end{array}$$

where *x*_ma_ is the model compensation term, *x*_*ms*_ is the robust feedback term, *K*_1_ = diag{*K*_11_, *K*_12_, *K*_13_} is the linear feedback gain, Γ_1_ > 0 is a diagonal adaptation rate matrix, *x*_msn_ is a nonlinear feedback item, *K*_*f*_ = *K*^−1^, and $K_{f}{\dot{x}}_{1d}-\Delta_{1n}=f_{\theta_{F}}+Y_{\theta_{F}}\theta_{F}$.

We can treat *x*_*m*_ as inferred human motion intent, and then, the desired joint positions can be obtained as

(37) $$\begin{array}{c} \begin{array}{c} q_{c1m}={\text{invkine}}_{\text{ub}}\left(x_{m}\right) \end{array}. \end{array}$$

Let *z*_2*h*_ = Kine(*x*_21_) − *x*_*m*_, where Kine means kinematics. Then, the first error dynamics is given as

(38) $$\begin{array}{c} \begin{array}{c} K_{f}{\dot{z}}_{1}=-K_{1}z_{1}-z_{2h}+\Delta_{1}+Y_{\theta F}{\tilde{\theta }}_{F}-x_{msn}. \end{array} \end{array}$$

Similar to [18], in order to obtain the desired motion trajectories (${\hat{q}}_{c1m}$, ${\hat{\dot{q}}}_{c1m}$, ${\hat{\ddot{q}}}_{c1m}$, and ${\hat{q}}_{c1m}^{\left(3\right)}$), a output differential observer is adopted.

#### 3.3.2. Low-Level MIMO Motion Tracking Controller

In low-level controller design, a motion tracking control algorithm making the position tracking error $z_{2}=x_{21}-{\hat{q}}_{c1m}$ converge to zero or to be bounded is proposed with the following design procedures.

**Step 1 .**

Specify the desired torque *τ*_actd_ for *B*_Dsp_*hP*_*L*_ that achieves accurate motion tracking (i.e., $x_{21}⟶{\hat{q}}_{c1m}$ if *B*_Dsp_*hP*_*L*_ = *τ*_*actd*_).

Treating *B*_Dsp_*hP*_*L*_ as the virtual control input in this part, the control law *τ*_actd_ is given as

(39) $$\begin{array}{c} \begin{array}{c} \tau_{\text{actd}}=\tau_{\text{actda}}+\tau_{\text{actds}},\\ \tau_{\text{actda}}=f_{0}+Y\hat{\beta }+Y_{B}{\hat{B}}_{\theta }-{\hat{\Delta }}_{3n}-J_{\text{ub}}^{T}F_{\text{hmub}},\\ \tau_{\text{actds}}=-K_{3s1}z_{3}+\tau_{\text{actdsn}},\\ z_{3}={\dot{z}}_{2}+K_{2}z_{2},\\ K_{3s1}=g_{3}{\left\|\Gamma_{2}\phi_{3}\right\|}^{2}+K_{3}, \end{array} \end{array}$$

where *τ*_actda_ is the term for model compensation, *τ*_actds_ is the robust feedback item, *K*_3*s*1_ is the gain for linear feedback, *K*_3_ > 0, *g*_3_ > 0, Γ_2_ > 0, and *τ*_actdsn_ is a term for nonlinear feedback. *Bx*_31_ = *Y*_*B*_(*x*_31_)*B*_*θ*_.

**Step 2 .**

Torque allocation (i.e., specify the desired load force *P*_Ld_ for each hydraulic cylinder such that *B*_Dsp_*hP*_Ld_ = *τ*_actd_).

With *τ*_actd_ given in Step 4, the next task is to figure out the desired load force for each hydraulic cylinder such that the combined effort equals *τ*_actd_. Since the system is overactuated, there is an infinite number of solutions unless additional constraints can be added. Here, an intuitive scheme inspired from the CGA data during double stance is used to allocate the operational force between the two legs without relying on computationally expensive optimization methods. It is observed that the leg with foot lying closest to the torso center of mass takes a greater portion of the load [3]. Thus, the following constraints are added:

(40) $$\begin{array}{c} \begin{array}{r} \frac{J_{\text{ubL}}^{-T}\tau_{L}}{J_{\text{ubR}}^{-T}\tau_{R}}=\frac{x_{TR}}{x_{TL}} \end{array}, \end{array}$$

where *J*_ubL_ and *J*_ubR_ represent the Jacobian matrix at the back point in the left leg and in the right leg, respectively. $\tau_{L}=\left[\begin{array}{ccc} \tau_{1} & \tau_{2} & \tau_{3} \end{array}\right]$. $\tau_{R}=\left[\begin{array}{ccc} \tau_{6} & \tau_{5} & \tau_{4} \end{array}\right]$. *x*_*TR*_ and *x*_*TL*_ represent the horizontal distance from back to right ankle and the horizontal distance from back to left ankle. With (40), *P*_*Ld*_ can be solved.

**Step 3 .**

Specify the desired flow*Q*_Ld_ for *Q*_*L*_ so that the actual load force tracks the desired load force synthesized in Step 5.

The same as [18], the joint velocity and acceleration used to compute ${\dot{P}}_{\text{Ld}}$ for adaptive model compensation are estimated through an adaptive robust observer.

Define the observer errors as

(41) $$\begin{array}{c} \begin{array}{c} e_{o1}=x_{21}-y,\\ \begin{array}{c} e_{o2}={\dot{e}}_{o1}+K_{o1}e_{o1}=x_{31}-{\dot{y}}_{r},\\ {\dot{y}}_{r}≜\dot{y}-K_{o1}e_{o1}. \end{array} \end{array} \end{array}$$

Then the nonlinear observer can be designed as

(42) $$\begin{array}{c} \begin{array}{c} e_{o1}=x_{21}-y,\\ \begin{array}{c} e_{o2}={\dot{e}}_{o1}+K_{o1}e_{o1}=x_{31}-{\dot{y}}_{r},\\ {\dot{y}}_{r}≜\dot{y}-K_{o1}e_{o1}, \end{array}\\ {\bar{M}}_{\text{Dsp}}{\ddot{y}}_{r}=B_{\text{Dsp}}{hP}_{L}+J_{\text{ub}}^{T}F_{\text{hmub}}-{\bar{C}}_{\text{Dsp}}{\dot{y}}_{r}-{\bar{G}}_{\text{Dsp}}-{\bar{B}\dot{y}}_{r}+{\bar{\Delta }}_{3n}+T_{\text{os}}+\left(K_{o2}+K_{o2s}\right)e_{o2},\\ {\dot{\bar{\theta }}}_{q}=-\text{Proj}\left(\Gamma_{o}\phi_{o}^{T}e_{o2}\right), \end{array} \end{array}$$

where *y* and ${\dot{y}}_{r}$ are the estimated joint positions and joint velocities, *K*_*o*1_ is any gain matrix, ${\bar{M}}_{\text{Dsp}}$, ${\bar{C}}_{\text{Dsp}}$, and ${\bar{G}}_{\text{Dsp}}$ represent the estimated matrices using new parameter estimation ${\bar{\theta }}_{q}$, *K*_*o*2_ is the gain for linear feedback, *K*_*o*2*s*_ is the gain for nonlinear feedback, and *T*_*os*_ is the robust feedback term.

Replacing ${\dot{q}}_{c1}$ with ${\dot{y}}_{r}$ in *P*_Ld_, the estimated *P*_Ld_ can be obtained where ${\hat{P}}_{\text{Ld}}=P_{\text{Ld}}\left(q_{c1},{\dot{y}}_{r},{\hat{\theta }}_{q},t\right)$. Let ${\hat{z}}_{4}=P_{L}-{\hat{P}}_{\text{Ld}}$. Treating *Q*_*L*_ as the control input in this part, the proposed control law making ${\hat{z}}_{4}=P_{L}-{\hat{P}}_{Ld}$ converge to zero or bounded is synthesized as follows:

(43) $$\begin{array}{c} \begin{array}{c} Q_{\text{Ld}}=Q_{\text{Lda}}+Q_{\text{Lds}},\\ Q_{\text{Lda}}=\frac{1}{{\hat{\beta }}_{e}}\left(-\phi_{4c}^{T}{\hat{\theta }}_{q}-{hB}_{\text{Dsp}}^{T}z_{3}+\frac{\partial {\hat{P}}_{\text{Ld}}}{\partial x_{2}}x_{3}+\frac{\partial {\hat{P}}_{\text{Ld}}}{\partial {\dot{y}}_{r}}{\ddot{y}}_{r}+\frac{\partial {\hat{P}}_{\text{Ld}}}{\partial t}\right),\\ Q_{\text{Lds}}=\frac{1}{\beta_{e\min}}\left(-K_{4s1}{\hat{z}}_{4}\right)+Q_{\text{Ldsn}},\\ K_{4s1}=g_{4}{\left\|\Gamma_{2}\phi_{4}\right\|}^{2}+d_{4}{\left\|\frac{\partial P_{Ld}}{\partial {\overset{\wedge }{\theta }}_{q}}\right\|}^{2}+K_{4}, \end{array} \end{array}$$

where *Q*_Lda_ and *Q*_Lds_ are the terms for model compensation and robust feedback, respectively, $\phi_{4c}={\left[\begin{array}{ccccc} \phi_{4c1} & \phi_{4c2} & \phi_{4c3} & \phi_{4c4} & \phi_{4c5} \end{array}\right]}^{T}$ is the vector with *ϕ*_4*c*1_ = 0_6×16_, *ϕ*_4*c*2_ = 0_6×6_, *ϕ*_4*c*3_ = 0_6×6_, *ϕ*_4*c*4_ = −*q*_*v*_*x*_3_, and *ϕ*_4*c*5_ = *I*_6×6_, *K*_4*s*1_ is the linear feedback gain, *K*_4_ > 0, *g*_3_ > 0, *d*_4_ > 0, and *Q*_Ldsn_ is a nonlinear feedback term.

Like ${\hat{\theta }}_{F}$, ${\hat{\theta }}_{q}$ is synthesized as

(44) $$\begin{array}{c} {\dot{\hat{\theta }}}_{q}=\text{Proj}\left[\Gamma_{\theta_{q}}\left(\phi_{3}z_{3}+\phi_{4}{\hat{z}}_{4}\right)\right], \end{array}$$

where Γ_*θ*_*q*__ > 0 is the adaptation rate matrix.

Ultimately, we can obtain the control voltage of the valves as

(45) $$\begin{array}{c} u_{i}=\frac{Q_{\text{Ldi}}}{k_{q1i}\left(A_{1i}/V_{1i}\right)\sqrt{\Delta P_{1i}}+k_{q2i}A_{2i}/V_{2i}\sqrt{\Delta P_{2i}}},i=\left\{1...6\right\}. \end{array}$$

### 3.4. Main Results

Furthermore, if Δ_*i*_ = 0,  *i* = 3, 4, after a finite time, zero final tracking error can be achieved; that is, *z*_2_⟶0, as *t*⟶∞.Furthermore, if Δ_1_ = 0 and ${\dot{K}}_{f}=0$ after a finite time, a bounded force tracking error can be guaranteed with integral converging to zero asymptotically, i.e., *z*_1_⟶0, as *t*⟶∞.

**Theorem 1 .**

For low-level motion tracking, if the control gains satisfy *λ*_min_(*K*_*o*2_) ≥ *k*_*o*2_, *λ*_min_(*K*_*o*2*s*_) ≥ 1/2*σ*_*e*_^2^, *λ*_min_(*K*_3_) ≥ *k*_3_ + 1/2, *λ*_min_(*K*_4_) ≥ *k*_4_, *g*_3_ > 2/4*d*_4_, and *g*_4_ > 2/4*d*_4_, bounded motion tracking errors and observer errors can be guaranteed by the control law (45), which is described by

(46) $$\begin{array}{c} \begin{array}{c} V_{s4}\left(t\right)\leq \exp\left(-\lambda t\right)V_{s4}\left(0\right)+\frac{\varepsilon }{\lambda }\left[1-\exp\left(-\lambda t\right)\right] \end{array}, \end{array}$$

where

(47) $$\begin{array}{c} V_{s4}=\frac{1}{2}\left(e_{o2}^{T}M_{\text{Dsp}}e_{o2}+z_{3}^{T}M_{\text{Dsp}}z_{3}+{\hat{z}}_{4}^{T}{\hat{z}}_{4}\right),\\ \lambda =2\min\left\{\frac{\lambda_{\min}\left(K_{3}\right)}{\sup_{t}\left\{\lambda_{\max}\left(M_{\text{Dsp}}\left(t\right)\right)\right\}},\frac{\beta_{e}}{\beta_{e\min}}\lambda_{\min}\left(K_{4}\right),\frac{\lambda_{\min}\left(K_{o2}\right)}{\sup_{t}\left\{\lambda_{\max}\left(M_{\text{Dsp}}\left(t\right)\right)\right\}}\right\},\\ \varepsilon =\varepsilon_{o}+\varepsilon_{3}+\varepsilon_{4}. \end{array}$$

**Theorem 2 .**

For human motion intent inference in high-level controller, if the zero tracking error *z*_2*h*_ = 0 is realized in the inner loop, a bounded human-machine interaction force tracking error can be guaranteed by the control law (36), which is described by

(48) $$\begin{array}{c} \begin{array}{c} V_{s1}\left(t\right)\leq \exp\left(-\lambda_{1}t\right)V_{s1}\left(0\right)+\frac{\varepsilon_{1}}{\lambda_{1}}\left[1-\exp\left(-\lambda_{1}t\right)\right] \end{array}, \end{array}$$

where

(49) $$\begin{array}{c} V_{s1}=\left(1/2\right)z_{1}^{T}K_{f}z_{1},\\ \lambda_{1}=2\frac{\lambda_{\min}\left(K_{1}\right)}{\sup_{t}\left\{\lambda_{\max}\left(K_{f}\left(t\right)\right)\right\}}. \end{array}$$

Theorems 7 and 8 can be proved using arguments in [18]. From Theorems 7 and 8, if large controller gains are selected, the force tracking error can still be small when the desired trajectory changes.

### 3.5. ARCFC Design for Left Leg Support

**Figure 4:**
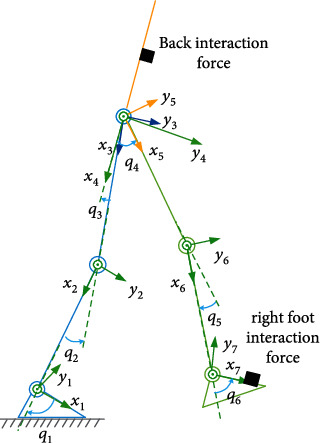
Human-machine interaction force minimized in left leg support. In this walking phase, six interaction force components at the back and right foot contact points will be minimized.

For the walking phase of left leg support, there exist six independent degree of freedoms and six control inputs, which can be seen from the dynamic model (7). Thus, we can control six independent human-machine interaction force components. Here, we select to minimize six interaction force components at the back and right foot, as shown in Figure 4.

The dynamics of left leg support is a serial chain one, and there is no overactuated characteristic in the system. Thus, the torque allocation is not needed in the controller design for left leg support. Other steps are almost the same as the ARCFC for double leg support except that the independent degrees of freedoms becomes six compared to three in double leg support. Here, for simplicity, the detail controller design process for left leg support is omitted in this paper.

## 4. Comparative Simulations

### 4.1. Simulation Setup

Based on the dynamic model, a simulation model is constructed in MATLAB/Simulink. The simulation parameters are the same as those in [18]. At first, the estimates of system parameters are set to be real values. The lumped disturbances are set to zero. The sampling time is selected as *t*_*s*_ = 0.0001*s*. The value for desired human-machine interaction force is set to zero. Considering the similarity between human body and lower limb exoskeleton, it is better to use human clinical gait analysis (CGA) data as desired joint position for simulation. However, the CGA data is different for people with different height and walking speed. Since the sinusoid curves are often used as desired trajectory for simulation of control algorithms. For a preliminary validation of the proposed controller performance, we choose the desired joint motion trajectory as sinusoid curves. The amplitude and the frequency of the sinusoid curves can be selected on the same order of magnitude as that of CGA data. In the simulation, the following control algorithms are conducted:

*C1*: The Low-Level PID Control with Velocity Feedforward

The control law is given by

(50) $$\begin{array}{c} \begin{array}{c} u=-K_{p}z_{2}-K_{I}\int_{0}^{t}z_{2}dt-K_{d}{\dot{z}}_{2}+V_{f}{\dot{x}}_{2id}, \end{array} \end{array}$$

In the simulation, a Z-N method with slight adjustments is used to obtain the control gains of PID controller. For left leg support, *K*_*p*_ = diag{10, 10, 10, 10, 10, 10}, *K*_*I*_ = diag{10, 10, 10, 10, 10, 10}, *K*_*d*_ = diag{30, 30, 20, 20, 15, 15}, and *V*_*f*_ = diag{0.1, 0.1, 0.1, 0.1, 0.1, 0.1}. For double leg support, *K*_*p*_ = diag{10, 10, 10, 10, 10, 10}, *K*_*I*_ = diag{10, 10, 10, 10, 10, 10}, *K*_*d*_ = diag{30, 30, 20, 20, 30, 30}, and *V*_*f*_ = diag{0.1, 0.1, 0.1, 0.1, 0.1, 0.1}.

*C2*: The Proposed Low-Level Motion Tracking Controller

According to the gain tuning rules described in [29], for left leg support, *K*_2_ = diag{40, 40, 40, 40, 40, 40}, *K*_3_ = diag{100, 100, 100, 100, 100, 100}, *K*_4_ = diag{100, 100, 100, 100, 100, 100}, *K*_*o*1_ = diag{40, 40, 40, 40, 40, 40}, and *K*_*o*2_ = diag{100, 100, 100, 100, 100, 100}. For double leg support, *K*_2_ = diag{320, 320, 320}, *K*_3_ = diag{800, 800, 800}, and *K*_4_ = diag{800, 800, 800, 800, 800, 800}, *K*_*o*1_ = diag{320, 320, 320}, *K*_*o*2_ = diag{800, 800, 800}. The adaptive rates are chosen to be zero for simplicity.

*FARC*: The High-Level Control Algorithms Proposed in This Paper

Different high-level controller gains are selected for different low-level controllers. In left leg support walking phase, for C1, the controller gains are *K*_1_ = diag{4, 4, 4, 4, 4, 4} and Γ_1_ = diag{0_1×6_, 0.2_1×2_, 2,0.2_1×3_}. For C2, the controller gains are *K*_1_ = diag{8, 8, 8, 8, 8, 8} and Γ_1_ = diag{0_1×6_, 0.25_1×2_, 4, 0.25_1×3_}. In double leg support walking phase, for C1, the controller gains are chosen as *K*_1_ = diag{4, 4, 4} and Γ_1_ = diag{0_1×3_, 1_1×3_}. For C2, the controller gains are *K*_1_ = diag{8, 8, 8} and Γ_1_ = diag{0_1×3_, 4, 15, 4}.

To show the control performance, three sets are simulated:

Set1: motion tracking control of two low-level controllers.

Set2: nominal interaction force control.

Set3: interaction force control to load change.

Set4: interaction force control to human-machine interface modeling errors.

### 4.2. Simulation Results

**Figure 5:**
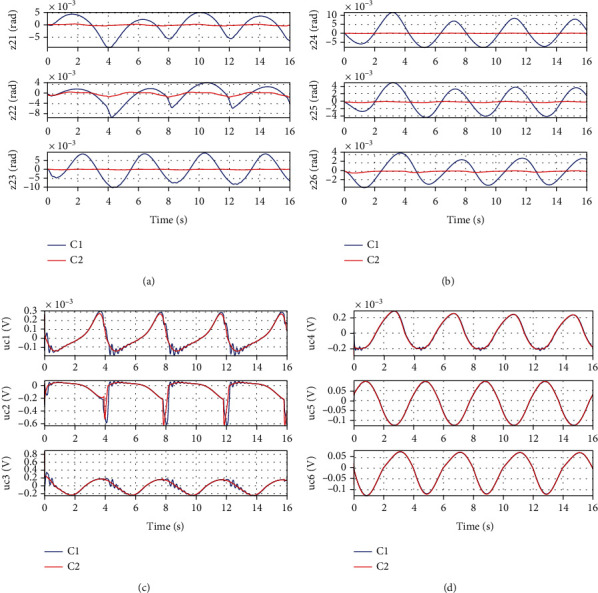
Simulation results of left leg support for Set1: (a) motion tracking error for the left leg joints, (b) motion tracking error for the right leg joints, (c) control input for the left leg joints, and (d) control input for the right leg joints.

**Figure 6:**
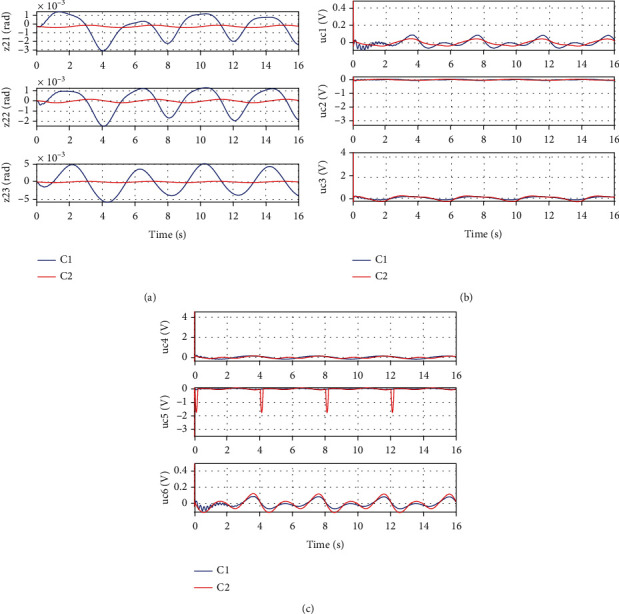
Simulation results of double leg support for Set1: (a) motion tracking error for left leg joints, (b) control input for left leg joints, (c) control input for right leg joints.

In *Set1*, the desired motion trajectory for left leg support is selected as *x*_2*d*_ = [−2 + 0.2sin((*π*/2)*t* − *π*/2), 0.5 + 0.2sin((*π*/2)*t* − *π*/2), −0.3 + 0.2sin((*π*/2)*t* − *π*/2), 0.3 + 0.2sin((*π*/2)*t* − *π*/2), −0.5 + 0.2sin((*π*/2)*t* − *π*/2), 2 + 0.2sin((*π*/2)*t* − *π*/2)]rad. The desired motion trajectory for double leg support is selected as *x*_2*d*_ = [−2 + 0.2sin((*π*/2)*t* − *π*/2), 0.5 + 0.2sin((*π*/2)*t* − *π*/2), 0.2sin((*π*/2)*t* − *π*/2)]rad. Figures 5 and 6 show the simulation results for Set1. It is seen that for both left leg support and double leg support, the proposed low-level controller (C2) achieves a smaller motion tracking error. The reason is that the controller gains of C1 can only be selected quite limitedly because of neglecting the strongly coupled dynamics in the controller design, while the controller gains of C2 can be selected as larger values due to the consideration of multi-joint coupling in the control algorithm design so as to achieve a better motion tracking performance.

**Figure 7:**
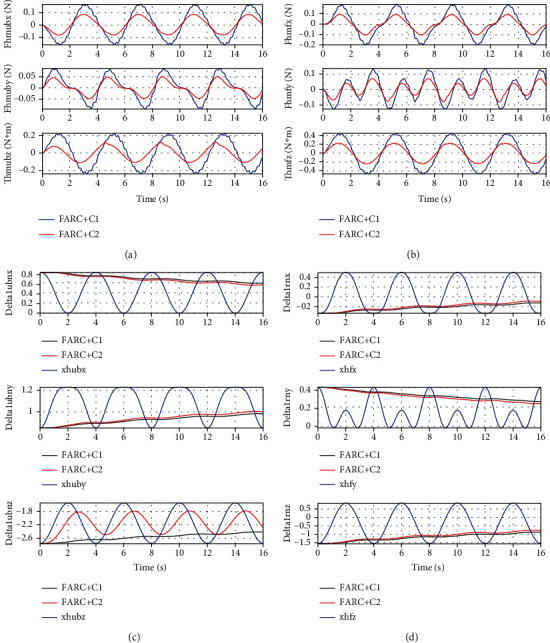
Simulation results of left leg support for Set2: (a) human-machine interaction force at the back, (b) human-machine interaction force at the right foot, (c) parameter estimation of Δ_1*n*_ at the back, and (d) parameter estimation of Δ_1*n*_ at the right foot.

**Figure 8:**
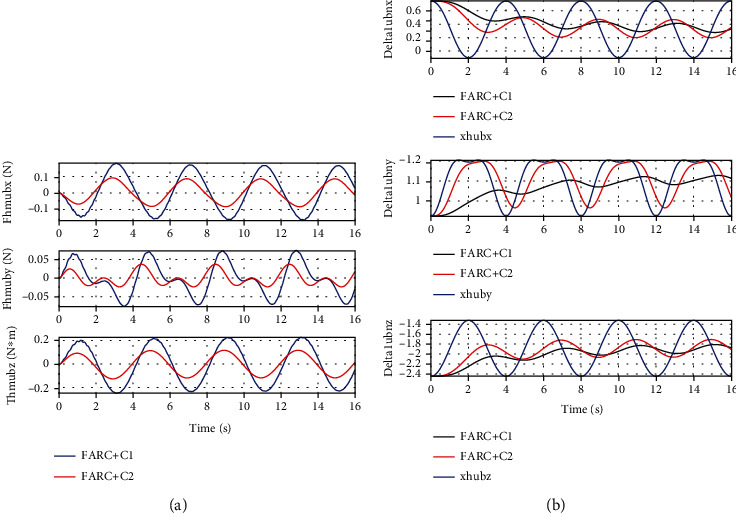
Simulation results of double leg support for Set2: (a) human-machine interaction force at the back, (b) parameter estimation of Δ_1*n*_ at the back.

For *Set2*, by passing the sinusoid curves *x*_2*d*_ = [−2 + 0.2sin((*π*/2)*t* − *π*/2), 0.5 + 0.2sin((*π*/2)*t* − *π*/2), −0.3 + 0.2sin((*π*/2)*t* − *π*/2),0.3 + 0.2sin((*π*/2)*t* − *π*/2), −0.5 + 0.2sin((*π*/2)*t* − *π*/2), 2 + 0.2sin((*π*/2)*t* − *π*/2)]rad through the kinematics equations for left leg support and passing the sinusoid curves *x*_2*d*_ = [−2 + 0.2sin((*π*/2)*t* − *π*/2), 0.5 + 0.2sin((*π*/2)*t* − *π*/2), 0.2sin((*π*/2)*t* − *π*/2)]rad through the kinematics equations for double leg support, the trajectory of human motion *x*_*h*_ can be finally solved. Without much performance compromised, only Δ_1*n*_ is selected to be adapted. With high-level controller selected as FARC, Figures 7 and 8 show that FARC+C2 achieve a smaller interaction force than that of FARC+C1. It is because a larger closed loop bandwidth can be achieved by C2 due to considering all characteristic of system dynamics, which results in larger high-level controller gains and adaptive rates. Then, better parameter estimation and force control performance can be achieved by FARC+C2.

**Figure 9:**
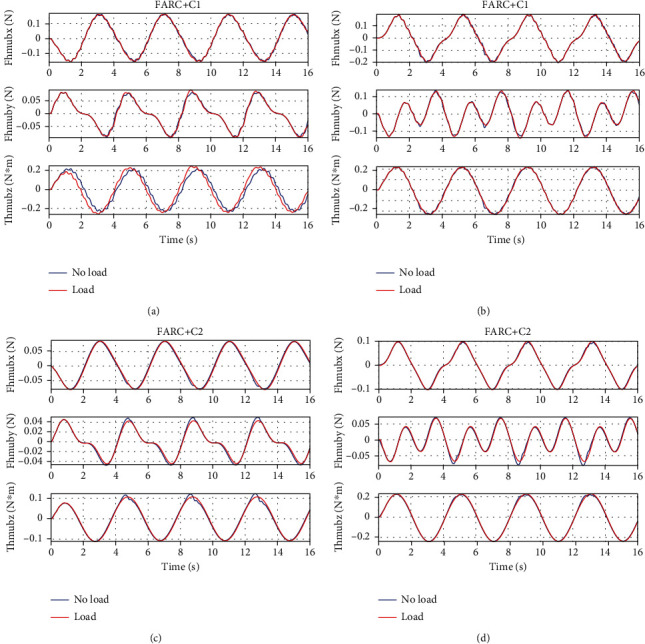
Human-machine interaction force of left leg support for Set3: (a) at the back for FARC+C1, (b) at the right foot for FARC+C1, (c) at the back for FARC+C2, and (d) at the right foot for FARC+C2.

**Figure 10:**
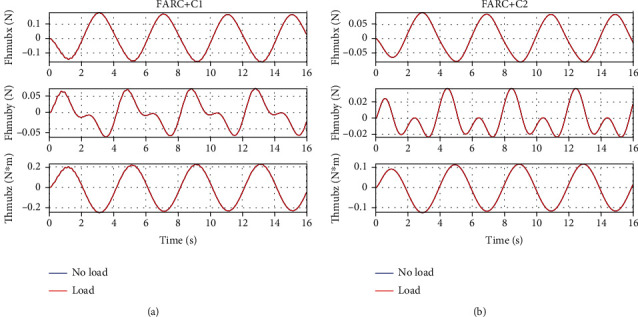
Human-machine interaction force of double leg support for Set3: (a) at the back for FARC+C1 and (b) at the back for FARC+C2.

In *Set3*, a 2.72*kg* weight is mounted at the shank to study the performance of the proposed ARCFC to parameter variation. From Figures 9 and 10, we can see that a consistent performance can be achieved both for the proposed ARCFC (FARC+C2) and the PID cascade force controller (FARC+C1) to load variation. Since the proposed ARCFC achieves higher closed loop bandwidth and faster parameter adaptation, model uncertainties due to load variation can be compensated more quickly and accurately which leads to a smaller interaction force and more consistent force control performance load variation.

**Figure 11:**
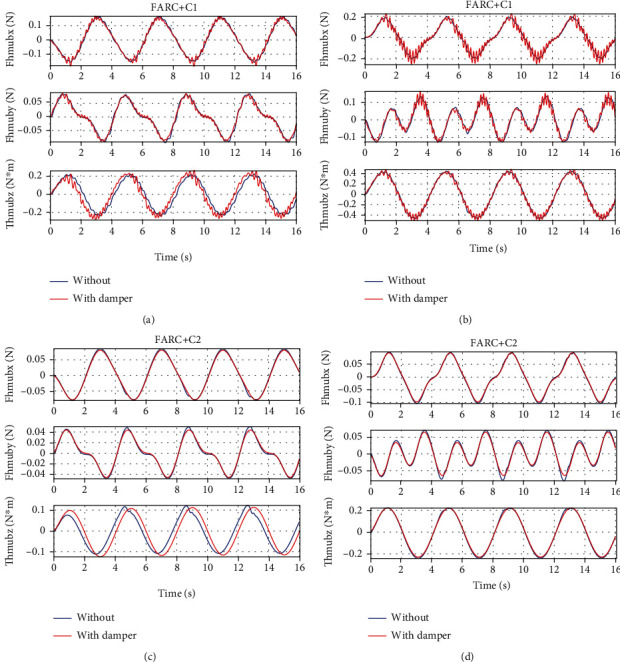
Simulation results of left leg support for Set4: (a) human-machine interaction force at the back for FARC+C1, (b) human-machine interaction force at the right foot for FARC+C2, (c) human-machine interaction force at the back for FARC+C2, and (d) human-machine interaction force at the right foot for FARC+C2.

**Figure 12:**
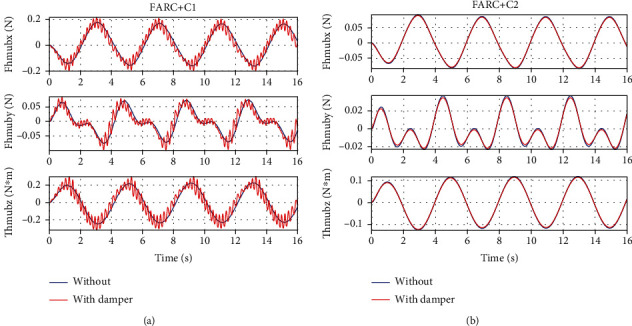
Human-machine interaction force of double leg support for Set4: (a) at the back for FARC+C1 and (b) at the back for FARC+C2.

In *Set4*, the human-machine interface dynamics is described as a spring-damper model which means in Equation (28), the modeling errors is described as ${\tilde{D}}_{1}=B_{\text{hm}}\left({\dot{x}}_{h}-{\dot{x}}_{e}\right)$ where *B*_hm_ is the damping ratio at the human-machine interface. From Figures 11 and 12, it can be seen that a consistent performance can be achieved for the proposed ARCFC (FARC+C2) to human-machine interface modeling errors both in left leg support phase and double leg support phase. For PID cascade force controller (FARC+C1), when adding the damping in the human-machine interface, the human interaction force becomes chattering. The reason is that the closed loop bandwidth and parameter adaptation rate of PID cascade force controller are limited leading to a poor disturbance rejection performance.

In this paper, only simulations are carried out, and also, the human motion trajectory is generated by sinusoid curves for a preliminary validation of the proposed controller performance. In the future, comparative experiments will be conducted on a real lower limb hydraulic exoskeleton platform to further validate the performance of the proposed interaction force controller in practical applications.

## 5. Conclusion

In this paper, a generalized multiphase dynamic modeling method is proposed for lower limb exoskeleton in which the dynamic model of each walking phase can all be obtained based on the dynamic model of a floating lower limb exoskeleton (with positions of the exoskeleton feet changed) and different holonomic constraints, which significantly simplify the dynamic modeling process of the multiphase lower limb exoskeleton. MIMO adaptive robust interaction force controllers with high level doing human motion intent inference while low level conducting human trajectory tracking are designed both for double leg support phase and single leg support phase. A torque allocation method is proposed to deal with the overactuated characteristic in double leg support. Comparative simulations show the effectiveness and better performance of the proposed multiphase human-machine interaction force controller. In our future research, we will do the modeling and controller design of underactuated lower limb exoskeleton systems. Stability analysis of uncontrolled internal dynamics and adaptive robust force control of underactuated exoskeleton systems will be conducted.

## Data Availability

The data that support the findings of this study are available from the corresponding author upon reasonable request.

## Supplementary Materials

Supplementary Materials Appendix: the description of each variable in this paper is shown in the following notation list.
