# Supplementary material for: Generalized Multiphase Dynamic Modeling and Precision Interaction Force Control of a Walking Lower Limb Hydraulic Exoskeleton
Source: Appl Bionics Biomech. 2022 Apr 7;2022:2801719. doi: 10.1155/2022/2801719 (PMC9076320; doi:10.1155/2022/2801719)
Supplement: Supplementary Materials — Appendix: the description of each variable in this paper is shown in the following notation list. [file 2801719.f1.docx]

Notation List

Variable Description

*q* General coordinates of a floating exoskeleton

*xl* , *yl* , *ql* Left foot positions of the exoskeleton

Variable Description

*qc*1*m* Desired joint position generated from high level controller

*xma* , *τactda* , *QLda* Model compensation terms

*xms* , *τactds* , *QLds* Robust feedback terms

*qi* , *i* = 1 *· · ·* 6 Angle of the ith joint

*Tact* Joint torque vector from the actuators

*xmsn* , *τactdsn* ,

*QLdsn*

Nonlinear robust feedback terms

*τi* , *i* = 1 *· · ·* 6 Actuation torque of the ith joint

*FL* , *FR* Ground contact force vectors

*Fxl* , *Fyl* , *Tl* Ground contact force components at the left foot *Fxr* , *Fyr* , *Tr* Ground contact force components at the right foot *M*, *C*, *G* System matrices and gravity force for a floating ex-

oskeleton

*Ba* Joint torque projection matrix for a floating ex- oskeleton

*JL* , *JR* , *JRq* Jacobian matrices

*Ki* , *Koi* Linear feedback gains

Γ1 , Γ*o* , Γ*θq* Adaptation rate matrices

*Cxl* , *Cyl* , *Cql* ,*Cxr* ,

*Cyr* , *Cqr*

Constant values

*qc* Joint position vector

*MLs p* , *CLs p* , *GLs p* System matrices and gravity force for left leg sup- port

*xr* , *yr* , *qr* Right foot positions of the exoskeleton

*MRs p* , *CRs p* , *GRs p* System matrices and gravity force for right leg sup- port

*qc*1 , *qc*2 Left leg and right leg joint position vector

*JRq*1 , *JRq*2 Jacobian matrix computed from *JRq*

*MDs p* , *CDs p* , *GDs p* ,

*BDs p*

*MH s* , *CH s* , *GH s* ,

*BH s*

System matrices, gravity force and joint torque pro- jection matrix for double leg support

System matrices, gravity force and joint torque pro- jection matrix for right heel strike

*V*1*i* , *V*2*i* , *βe* Total volumes of the two chambers in cylinder i and effective bulk modulus

*P*1*i* , *P*2*i* , *A*1*i* , *A*2*i* Absolute pressures and acting areas of the two chambers in cylinder i

*xLi* , *Q*1*i* , *Q*2*i* Displacement, Supply and return flow of the cylin- der i

*D*˜ 1 , *D*˜ 2 , *D*˜ 31*i* , *D*˜ 31*i* Lumped modeling errors and uncertainties

*kq*1*i* , *kq*2*i* Flow gain coefficients for the two loops of cylinder i

*xvi* , *ui* Spool displacement and control input of valve i

*Ps* , *Pr* Supply pressure and reference pressure *Fhm* Human machine interaction force vector *K* stiffness of the human-machine interface

*xh* , *xe* Human position and exoskeleton position at the con- tact point

*Fhmub* , *Fhmr* Interaction force vector at the back and right foot contact point

*xhub* human position at the back contact point

*xeub* , *xer* Exoskeleton position at the back and at right foot contact point

*invkineub* ,

*invkiner*

Inverse kinematics at the back and at right foot con- tact point

*x*1*d* Desired trajectory to be tracked by x1

*xm* Virtual control law in high level controller

1
